# The Expanding Family of Virophages

**DOI:** 10.3390/v8110317

**Published:** 2016-11-23

**Authors:** Meriem Bekliz, Philippe Colson, Bernard La Scola

**Affiliations:** 1 Aix-Marseille University, URMITE UM 63 CNRS 7278 IRD 198 INSERM U1905, 27 Boulevard Jean Moulin, 13385 Marseille CEDEX 05, France; meriembekliz@hotmail.com; 2 IHU Méditerranée Infection, Pôle des Maladies Infectieuses et Tropicales Clinique et Biologique, Fédération de Bactériologie-Hygiène-Virologie, Centre Hospitalo-Universitaire Timone, Méditerranée Infection, 264 rue Saint-Pierre, 13385 Marseille CEDEX 05, France; 3 Pôle des Maladies Infectieuses, Assistance Publique-Hôpitaux de Marseille and Aix Marseille Universite, URMITE, UM63, CNRS 7278, IRD 198, INSERM 1095, Faculté de Médecine, 27 Boulevard Jean Moulin, 13385 Marseille CEDEX 05, France

**Keywords:** virophages, mimivirus, *Megavirales*, metagenomic, mobilome

## Abstract

Virophages replicate with giant viruses in the same eukaryotic cells. They are a major component of the specific mobilome of mimiviruses. Since their discovery in 2008, five other representatives have been isolated, 18 new genomes have been described, two of which being nearly completely sequenced, and they have been classified in a new viral family, *Lavidaviridae*. Virophages are small viruses with approximately 35–74 nm large icosahedral capsids and 17–29 kbp large double-stranded DNA genomes with 16–34 genes, among which a very small set is shared with giant viruses. Virophages have been isolated or detected in various locations and in a broad range of habitats worldwide, including the deep ocean and inland. Humans, therefore, could be commonly exposed to virophages, although currently limited evidence exists of their presence in humans based on serology and metagenomics. The distribution of virophages, the consequences of their infection and the interactions with their giant viral hosts within eukaryotic cells deserve further research.

## 1. Introduction

Formerly, viruses were considered to be small particles with the ability to pass through 0.2 μm pore filters and remain invisible under a light microscope, in contrast to microbes [1]. The concept of the virus was unraveled in 1957 by André Lwoff, who mainly defined them on the basis of negative criteria. They were subsequently described as harboring small genomes with a limited number of genes, and devoid of translational apparatus components [1]. During the 20th century, viruses became increasingly established as small entities that need living cells to replicate, and were consecutively described as obligate parasites of eukarya, then bacteria and archaea [2], while some were discovered as causative agents of disease.

In 2003, the *Acanthamoeba polyphaga mimivirus* (APMV) was the first giant virus of amoeba discovered [3]. Its particle and genome sizes were approximately two to three times greater than for other viruses and similar to those of some bacteria, archaea, or eukarya microorganisms [3,4]. Mimivirus was the founder of a new viral family officially recognized by the International Committee on Taxonomy of Viruses (ICTV) and named *Mimiviridae*. Since then, dozens of mimivirus relatives have been described. Furthermore, other giant viruses have been discovered, either founding a new viral family, *Marseilleviridae*, or representing new putative viral families, including pandoraviruses [5,6], pithoviruses [7], faustoviruses [8], and *Mollivirus sibericum* [9]. These viruses were isolated using co-culture strategies on *Acanthamoeba* spp. for the most part, or *Vermamoeba vermiformis*. They were linked to a monophyletic group of double-stranded DNA (dsDNA) viruses (the nucleocytoplasmic large DNA viruses (NCLDVs)) described earlier, in 2001, comprising poxviruses, asfarviruses, ascoviruses, iridoviruses, and phycodnaviruses [10]. Giant amoeba viruses have dsDNA genomes larger than 360 kilobase pairs (kbp) and ranging up to over 2.5 megabase pairs (mbp), which harbor hundreds of genes [11,12,13]. In addition, some of these genes are unique among viruses, including some, the most emblematic, that encode for translation components, or others that are involved in DNA repair or amino acid metabolism, and a large proportion of the predicted genes are ‘ORFans’, as they are not found in any other organisms. Thus, giant amoeba viruses differ significantly from other viruses, while they share many properties with microbes from the three cellular domains of life. A major difference with these microbes remains the fact that they lack ribosomes. Based on their unique phenotypic and genotypic features, it was proposed in 2012 to reclassify viral families comprised of giant amoeba viruses, as well as NCLDV, in a new viral order, the *Megavirales* [14]. Thereafter, phylogenomic analyses led to proposing that these viruses may comprise a fourth branch of microbes or “TRUC” (Things Resisting Uncompleted Classification), which is still hotly debated [15,16].

In addition to the astounding discovery of giant amoeba viruses, mimivirus study led to the discovery of a smaller, albeit completely new type of virus. Indeed, in 2008, a small virion, named Sputnik, was spotted together with *Acanthamoeba castellanii mamavirus*, another mimivirus isolate. Sputnik was able to infect the mamavirus factory, which was harmful for this giant virus [17]. Thereby, it resembled bacteriophages by the fact that it impaired the replication of this virus while infecting its factory, which led to naming it a ‘virophage’, and it was later found that they could integrate in the large genome of mimiviruses [17,18]. It could be considered as the first virus infecting another virus, after the successive discoveries since the 1890s of viruses of eukaryotes, bacteria, and archaea. The discovery of the virophage amplified the revolution initiated by giant viruses in virology [19] and raised considerable interest in the field of evolutionary biology [2,4,20,21,22].

This review describes findings from studies conducted on virophages over the eight past years.

## 2. Virophage Discovery and History

Until 2008, *A. polyphaga mimivirus* remained the unique member of the family *Mimiviridae*, until the discovery of a second giant virus, named *A. castellanii mamavirus* that was isolated from cooling tower water collected in ‘Les Halles’, Paris, France [23,17]. At the same time, on electron microscopy, small virions with a diameter of approximately 50 nm were observed in the cytoplasm of mamavirus-infected *Acanthamoeba* cells [17]. These small viruses, named ‘Sputnik’, were present in the mamavirus factories, and their presence was associated with abnormal morphologies of mamavirus virions and a 70% reduction in infectious mamavirus progeny. The replication of Sputnik viruses was strictly dependent on the co-infection with a mimivirus into an acanthamoeba. The fact that they could use the factory of mimiviruses for their own propagation and were deleterious for the replication of these giant viruses made them functionally analogous to bacteriophages, and they were, hence, named virophages [17,24].

Since the discovery of this first virophage in 2008, others have been described, albeit mostly from metagenomic datasets (Table 1) [17]. Six close relatives of virophages were isolated from distinct locations, in France, Texas, Brazil, and Tunisia, and from various sources, including water, soil, and a contact lens rinse fluid (Table 1) [19,17,18,25,26,27,28]. The other virophages are known only by their genome, as a result of screening several metagenome sequences communities in various environments (Table 1). All of them have similar features among themselves, besides the difference in their size (17–30 kbp), their gene repertoire, and host viruses.

## 3. Morphological Features of Virophages

All isolated virophages are non-enveloped viruses with small icosahedral capsids approximately 35–74 nm in diameter. Three-dimensional structural studies have only been carried out on Sputnik virophages, using cryo-electron microscopy [35]. They showed that virions have a diameter of 74 nm and a hexagonal surface lattice with a T = 27 icosahedral capsid composed of 260 pseudohexameric and 12 pentameric capsomers located at the vertices. Pseudohexameric capsomers are built by trimerisation of monomers with a double jelly-roll fold. They have a diameter of approximately 7.5–8.5 nm, and fibers that are 5.5 nm-long protrude from their center. Pentameric capsomers have central cavities that could serve for DNA entry or exit, as in bacteriophages. A 4 nm-thick lipid bilayer was observed beneath the capsid shell.

## 4. Replicative Cycle of Virophages

**Figure 1:**
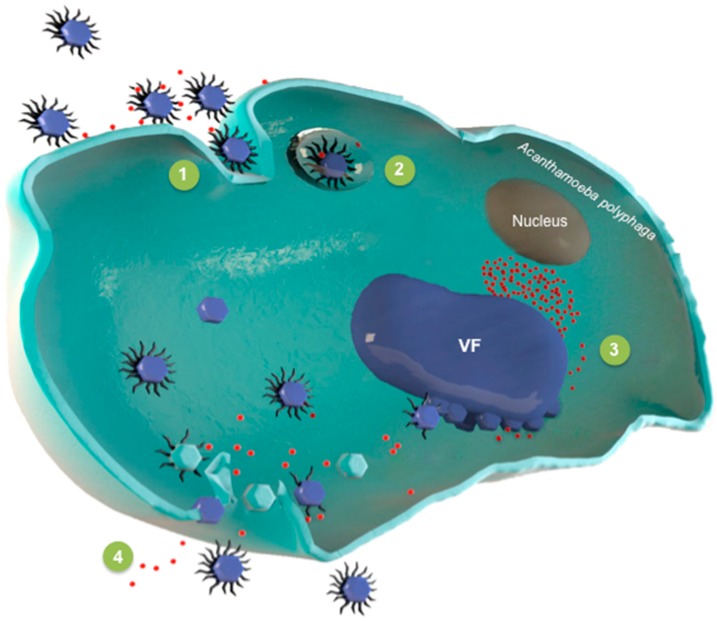
The intracellular life cycle of virophages: This scheme summarizes the different steps of the intra-amoebal life cycle of a virophage and its giant viral host. In (1), the giant virus and the virophage enter the amoeba; In (2), the giant virus and the virophage are internalized in vacuoles; The virus factory (VF) appears in (3), followed by the production of virophages and giant virus virions. Finally, the amoeba is lysed and the two types of viruses are liberated (4).

The replicative cycle of virophages, including its effects on the host virus, has only been described for Sputnik, in association with mamavirus [17,36]. The replication of Sputnik depends strictly on its viral host, and it only occurs within the giant viral factory [17,36]. Thus, Sputnik, alone, is unable to infect amoebas (Figure 1). It is suspected that due to its small size, it is not phagocytized by amoebas.

### 4.1. Entry of Sputnik in the Amoeba

The specific strategies concerning how the virophage can enter eukaryotic cells and replicate in its host giant virus factory are still not clear. Shortly before their entry into amoebae, virophages were observed attached to fibrils present on the surface of the giant virus, and it has been hypothesized that these fibrils were, therefore, essential to virophage entry [35,36,37]. In addition, mimivirus protein R135, which was associated with fibers [38], was found in the Sputnik protein set identified by proteomics [17], and mimivirus variants devoid of fibrils became resistant to infections by the virophage [37]. Thus, virophages may be internalized into amoebae together with mimiviruses through phagocytosis.

### 4.2. Virophages Invade the Giant Virus Factory

Between one and two hours post infection (h.p.i.), endocytic vacuoles can be observed in the amoebal cytoplasm. Then, an eclipse phase occurs that lasts approximately 2–4 h. During the eclipse phase, genome replication, transcription, and translation initiate. Both Sputnik and mimivirus replication occur in clearly-defined dense cytoplasmic areas, apart from the nucleus of the amoeba, named viral factories, which are centered around a small spherical compartment. At this stage, it is not possible to observe and isolate a virophage particle.

### 4.3. The Spread of Progeny Virophages

After the eclipse phase, the viral factory expands, and virophage progeny virions begin being produced at one pole of the giant viral factory, before the production of mimivirus virions [17]. Occasionally, different viral factories can be seen: one with virophage progeny and the other one producing mimivirus [36]. Around 16 h.p.i., all infected amoebae are completely filled with newly formed virophage and mimivirus particles. Free virophage virions can be detected in the cytoplasm or can accumulate within amoebal vacuoles. At 24 h.p.i., more than two thirds of the co-infected amoebae have been lysed, thus liberating the whole virophage progeny.

### 4.4. Replicative Cycle of Other Virophages

#### 4.4.1. Zamilon

The Zamilon virophage is selective and propagates only when associated with mimiviruses from lineages B or C [27]. Its replication occurs in the mimivirus factory. The co-infection of amoebae with Zamilon and mimiviruses does not appear to interfere with mimivirus propagation nor amoebal lysis by mimiviruses alone.

#### 4.4.2. Mavirus

The mavirus virophage replicates in *Cafeteria roenbergensis*, a marine phagotropic grazer, in the virus factory of the only giant virus known to infect this eukaryote, *Cafeteria roenbergensis virus* (CroV). Transmission electron microscopy shows the mechanism of entry of mavirus, which differs from that of Sputnik due to the fact that mavirus is endocytosed independently of CroV, most likely via the clathrin-mediated endocytosis pathways [19]. As in Sputnik with mimiviruses, mavirus inhibits the production of new CroV particles, and increases *Cafeteria roenbergensis* survival.

## 5. Genomic Structure and Features of Virophages

Currently, 18 full or partial genomes of virophages are available in the GenBank database (Table 1).

### 5.1. Genomic Organization

#### 5.1.1. Isolated Virophages

Some of the virophages have been isolated with their giant virus host using co-culture on eukaryotic cells they co-infect.

##### ●  Sputnik isolates

Since the discovery of the first Sputnik virophage in 2008, three other closely-related Sputnik-virophages have been isolated. Sputnik 2 was described in 2012 with lentillevirus, a mimivirus belonging to lineage A. It was isolated from contact lens storage case liquid of a 17-year-old myopic woman presenting with keratitis [39] (Table 1). Surprisingly, the genome of lentillevirus was found to contain an integrated genome of virophage Sputnik 2, as a ‘provirophage’, and previously unknown mobile genetic elements, which were named transpovirons. Sputnik 3 was detected by PCR in 2013 from a soil environmental sample with a mimivirus of lineage C that failed to grow in amoeba. However, Sputnik 3 was isolated independently of this giant virus from a co-culture procedure using mamavirus as a reporter. The Sputnik 1, 2, and 3 virophages are small icosahedral, non-enveloped viruses approximately 70 nm in diameter [17,18,26]. Sputnik Rio Negro, whose particle diameter is about 35 nm, was isolated in 2014 in association with Samba virus, a mimivirus of lineage A.

Sputnik virophages replicate in the presence of mimiviruses of *Acanthamoeba*, and they lead to abnormal virions and decreased infectivity and lytic ability of these mimiviruses. All Sputnik isolates have circular dsDNA genomes. These genomes are made of 18,343 bp for Sputnik (NC_011132), 18,338 bp for Sputnik 2 (NC_023846), and 18,338 bp for Sputnik 3 (NC_023847); the Sputnik Rio Negro genome is not yet available. The Sputnik 1, 2, and 3 genomes differ from each other by fewer than 10 base pairs and they have a low G + C content, as in mimiviruses [17,18,26,39]. They contain 20–21 open reading frames (ORFs) that encode proteins with a size of 88–779 amino acids. Four genes are homologous to eukaryotic and bacteriophages genes, three to mimivirus genes and one to an archaeal virus gene. The remaining 13 ORFs are ORFans, as they do not have any detectable homologues in sequence databases. This mosaic gene content suggests involvement in lateral gene transfers between viruses [17].

##### ●  Mavirus

Mavirus was the second virophage discovered [19]. Its virion is spherical and 60 nm in diameter. It was isolated in 2010 from coastal waters off Texas, USA. It depends for its propagation on CroV [25,40], which infects a marine bi-flagellate heterotrophic eukaryote [19,41]. Mavirus has a circular dsDNA genome of 19,063 bp (NC_015230) encoding 20 ORFs [19] with an average length of 883 nucleotides and a high A + T content. Ten ORFs showed sequence similarity to proteins from retroviruses, bacteria, eukaryotes, and dsDNA viruses, including four ORFs found in the Sputnik genome. Thus, Mavirus and Sputnik have homologous genes encoding a capsid protein, a predicted cysteine protease, a predicted GIY-YIG endonuclease/Zn-ribbon protein and a predicted DNA-pumping ATPase. A first evolutionary scenario that was proposed following the mavirus characterization was that Polintons originated from virophages [19], but the most recent scenario is that virophages are descendants of Polintons [42].

##### ●  Zamilon

Thereafter, Zamilon (‘neighbor’ in Arabic) was co-isolated in 2013 from Tunisian soil with the Mont1 virus, which belongs to the lineage C of *Mimiviridae* [27]. Its virion has a spherical shape and a diameter of approximately 50–60 nm. Zamilon grew well when the amoebae were infected with mimviruses of lineages B or C, but not with mimiviruses of lineage A. Additionally, it did not seem to affect the growth of the associated giant virus, its lytic ability, or cause the production of virions with abnormal morphology, contrary to Sputnik. The Zamilon genome is a circular dsDNA genome of 17,276 bp (NC_022990) with a low G + C content (29.7%) and contains 20 ORFs ranging from 222 bp to 2337 bp in length. It has only 76% nucleotide identity and 75% coverage with the Sputnik genome. Seventeen ORFs have significant homology with Sputnik ORFs (with 31%–86% identity), two ORFs have significant homology with *Megavirus chiliensis* (identity, 50%–67%) and one ORF has significant homology with a *Moumouvirus monve* ORF (identity, 72%) [27]. Moreover, phylogenetic analysis showed that Zamilon ORF19 had greater homology with lineage B and C mimiviruses than with lineage A mimiviruses and Sputnik virophages.

#### 5.1.2. Metagenomics and Genomics Detection

Several metagenomes have been investigated in previous studies and screened for the presence of virophages and *Megavirales* sequences. A total of 13 complete and partial genomic sequences of virophages were assembled based on the metagenomic sequences generated from Organic Lake in Antarctica, Dutch coastal waters, Yellowstone Lake, Ace Lake, a bioreactor, Qinghai Lake, and Dishui Lake. However, in most cases, the cellular and viral hosts for these virophages are unknown.

##### ●  Organic Lake Virophage (OLV)

The third virophage, Organic Lake virophage (OLV), was the first virophage detected in 2011 by metagenomics, from a sample collected in Organic Lake, a hypersaline meromictic lake in Antarctica [28]. In this lake, the layers of water have remained unmixed for years, decades or more. Virophage-like spherical particles with a diameter of 50 nm were observed by transmission electron microscopy. Other virion-resembling particles with a diameter of approximately 150–200 nm were also observed, and phylogenetic analyses first classified sequences from this lake as linked to phycodnaviruses, and subsequently as belonging to distantly related mimiviruses [43]. OLV has a circular dsDNA genome of 26,421 bp with a G + C content of 36.5% and was predicted to encode 24 proteins, which can have 27%–42% amino acid identity with Sputnik proteins. Six proteins are homologous to Sputnik proteins, including the capsid protein, the DNA packaging ATPase protein, the putative DNA polymerase/primase, and three proteins of unknown function.

##### ●  Phaeocystis globosa virophage

In 2013, analysis of metagenomes generated from Dutch coastal waters (southern North Atlantic and the North Sea) revealed the presence of a virophage-like genome. The assembly of Phaeocystis globosa virus strain PgV-16T (PgV-16T), a distantly related mimivirus, generated a 19,527-bp long scaffold in greater copy number than the PgV-16T virus corresponding to a virophage genome. Sixteen predicted ORFs were found in its genome but most of them had no significant matches in sequence databases. Three ORFs had homologs in mavirus, including an endonuclease, the predicted DNA primase and polymerase and one ORF had a homolog in the OLV virophage. The *Phaeocystis globosa* virophage may have lost structural genes, apart from a distant version of the major capsid protein (MCP) encoding gene [44]. No virion smaller than 75 nm in diameter was observed by electron microscopy in infected *P. globosa* culture cells. Santini et al. suggested that this element replicates as a provirophage integrated in the genome of its host virus or as a linear plasmid [29].

##### ●  Ace Lake Mavirus (ALM)

In 2013, a nearly complete genomic sequence from a virophage, named Ace Lake mavirus (ALM), was retrieved from an Ace Lake sample in Antarctica [30]. It was 17,767 bp in length, had a G + C content of 26.7%, and encoded 22 ORFs, among which 14 were homologous to mavirus ORFs.

##### ●  Yellowstone Lake virophages (YSLVs)

In 2013 and 2015, seven additional complete virophage genomic sequences (YSLV1-YSLV7) were obtained from Yellowstone Lake metagenomes. This suggested a substantial diversity of giant viruses and the eukaryotic hosts in this environment [30,31]. These genomes had a size ranging between 22 and 29 kbp.

##### ●  Hybrid virophages

Recently, Yutin et al. detected sequences of virophages corresponding to MCP from several metagenome datasets [45]. Thus, 16 MCP from putative virophages were identified in six environmental metagenomes generated from freshwater sediment, marine water, wastewater, activated sludge, rumen gut, and a bioreactor [45]. Phylogeny reconstruction showed that MCP from activated sludge and bioreactor metagenomes were clustered with Sputnik and Zamilon virophage MCP, whereas those from virophages of the Sargasso Sea metagenome were clustered with Organic Lake and Yellowstone Lake virophages. Sequences corresponding to mimiviruses were concurrently found, suggesting that this new family may parasitize these giant viruses. These findings also represented an additional case of detection of a virophage sequence from an animal, after the detection of 65 and 11 virophage-related sequences in human gut and animal-associated habitats, respectively [30]. In addition, in the study of Yutin et al. [45], phylogenetic analyses showed that virophage-related MCP found in the metagenome generated from sheep rumen belong to a new putative lineage of virophages, and assembly of metagenomic reads unexpectedly reconstructed a virophage-polinton hybrid genome.

##### ●  Zamilon 2

In 2015, the assembly of the partial genome of a Zamilon-like virophage was described from a non-aerated bioreactor metagenome, in which Yutin et al. concurrently detected Zamilon-related capsids sequences [45]. Comparative genomics and phylogenetic analyses indicated that the partial genome was that of a Zamilon-like virophage, named Zamilon 2 [32].

##### ●  Virophage sequences integrated in *Bigelowiella natans* genomes

In 2015, virophage genomes integrated into the nuclear genome of the unicellular alga *Bigelowiella natans* were described, and found to be highly transcribed [46]. In the same genome, they also observed sequences from *Megavirales* members and repeated elements that matched with transpovirons. Based on these findings, it was suggested that the genome of these chlorarachniophytes could have recorded genetic prints of virophages to use them as a molecular weapon against giant viruses, conferring a sort of immunity.

##### ●  Dishui Lake Virophage (DSLV1)

A novel group of virophages was discovered in 2016 from Dishui Lake, an artificial freshwater lake in Shanghai, China. A complete genomic sequence from this virophage, named Dishui Lake virophage (DSLV1), was obtained [33]. Its assembly generated a circular dsDNA genome of 28,788 bp with a G + C content 43.2%, which harbors 28 ORFs. Other metagenomic sequences from the same Dishui Lake sample were related to virophages and phycodnaviruses. These metagenomic sequences were closely related to YSLVs and OLV.

##### ●  Qinghai Lake Virophage (QLV)

In 2016, another new virophage was described from a metagenome of a planktonic microbial community of Qinghai Lake, a Tibetan mountain lake. A complete genomic sequence from this virophage, named Qinghai Lake virophage (QLV), was obtained, which contains 23,379 bp, has a G + C content of 33.2%, and encodes 25 ORFs [34]. QLV shares between seven and 11 homologous genes with YSLVs and OLV. Eleven ORFs were not found in other virophages, thus being specific to QLV. Phylogenetic analyses showed a closer evolutionary relationship between QLV, OLV, and YSLVs than with Sputnik and mavirus. In the same metagenome, Oh et al. identified sequences closely related to phycodnaviruses, suggesting that QLV are associated with these viruses [34].

##### ●  Virophage capsid encoding sequences found in metagenomes

Many virophage capsid homologs were detected in different metagenomic datasets generated from microbial communities sampled in lakes, rivers, Antarctic lakes, and freshwater lagoons, (Yellowstone, Lanier, Qinghai, Mendota, Trout, Erken, Vattern, Ekoln, Damariscotta, Spark, Amazon, Albufera). They are also detected from the metavirome of distinct Antarctic hyperarid desert soil communities, activated sludge metagenome, freshwater sediment metagenome, gut metagenome, marine metagenome, and wastewater metagenome, testifying to the widespread occurrence of virophages in the biosphere [45,47]. Taken together, there is an expanding diversity of virophages.

### 5.2. Conserved and Core Genes

**Figure 2:**
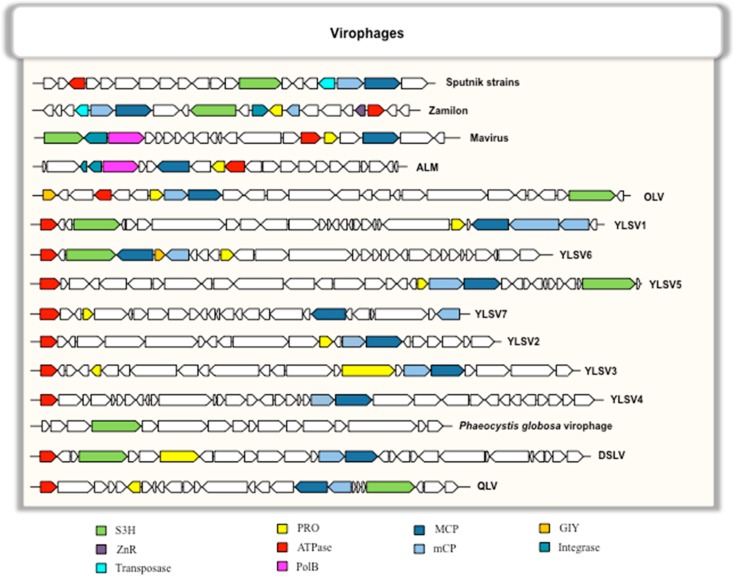
Comparison of conserved gene architectures in the genomes of virophages. MCP: major capsid protein; mCP: minor capsid protein; S3H: DNA helicase/primase; ZnR: zinc-ribbon domain; PRO: cysteine protease; ATPase: putative FtsK-HerA family DNA packaging ATPase; polB: family B DNA polymerase; GIY: GIY-YIG endonuclease/Zn-ribbon.

Among the 20–34 ORFs present in virophage genomes, six genes are conserved in most or all virophages and are considered as virophage core genes [48]. These six genes encode a MCP, a minor capsid protein (mCP), a putative FtsK-HerA family DNA packaging ATPase (ATPase), a cysteine protease (PRO), a DNA helicase/primase (S3H), and a zinc-ribbon domain containing protein (ZnR) encoded by a gene present in most, but not all, virophages (Figure 2) [48]. In addition, in several virophages, the set of conserved genes encodes integrases of two different families (a putative tyrosine integrase found in Sputnik [17] and a putative rve integrase found in ALM and mavirus) [19,30]. The existence of these core genes suggests a monophyletic origin for virophages, which supported the creation of a new viral family, named *Lavidaviridae*, for virophages, which includes Sputnik, Zamilon and mavirus isolates [48].

### 5.3. Phylogeny of Virophages

**Figure 3:**
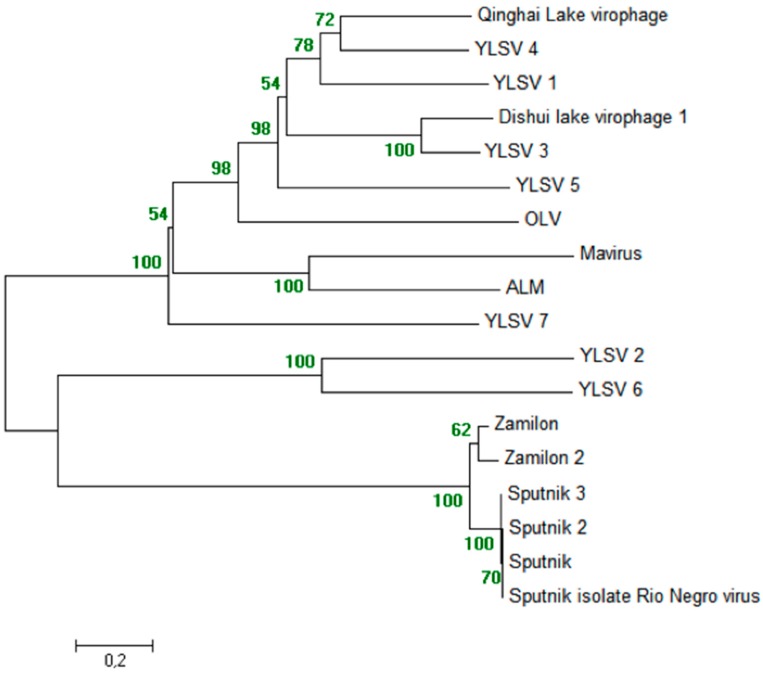
Phylogeny reconstruction based on the capsid proteins from virophages. The sequences were aligned with the Muscle software [49]; the un-rooted tree was built using the maximum likelihood method.

In a phylogeny reconstruction based on the MCP of all known virophages, all Sputnik and Zamilon virophages form a robustly supported monophyletic group (bootstrap value, 100%). In addition, mavirus is clustered with ALM, DSLV1 is clustered with YSLV3, and YLSV2 is clustered with YSLV6, with high reliabilities (bootstrap values, 100%) (Figure 3).

## 6. Classification of Virophages, New Agents in the Field of Virology

The diversity of virophages has considerably expanded during the past eight years. A debate on the classification of virophages as a satellite virus occurred [50,51,52]. The main argument in favor of a classification as a satellite was the fact that isolated virophages cannot multiply without its associated giant virus, but virophages were claimed to be more than just satellites. Recently, Krupovic et al. proposed creating the family *Lavidaviridae* (Lavida is for LArge VIrus-Dependent or Associated virus) for virophages, which has been officially recognized by the ICTV [48].

## 7. Virophages as Part of the Mobilome Associated to Giant Viruses

Virophages are a component of the mobilome of mimiviruses, together with self-splicing introns and transpovirons [18]. They can integrate, as provirophages, in mimivirus genomes, and may be involved in sequence exchanges between giant viruses and other microbes inside amoebae [16,18]. Strangely, the mavirus virophage shows an evolutionary relationship with large virus-like transposons of the Polinton superfamily. Polintons are widespread in eukaryotes and are widely found in various protists, suggesting their very ancient origin [42,53]. It was also suggested that they reside in the eukaryotic host nucleus [42]. Long inverted repeats are present in the mavirus genome that resemble those found in Maverick or Polinton transposable elements, further suggesting their evolutionary relationship. Mavirus and Sputnik virophages differ with respect to their genetic similarity with these transposons. The existence of an evolutionary connection between eukaryotic transposons of the Polinton family, virophages, and other viruses has been inferred [42,53]. The sympatric and parasitic lifestyle of mimiviruses and virophages inside amoeba cells seems to favor genetic innovation, with frequent DNA exchanges mediated by a complex mobilome [17,18].

## 8. MIMIVIRE, a Defence Mechanism against Virophages

Since the discovery of the first giant virus, several other mimiviruses have been isolated from different environments [54,55,56]. Pagnier et al. described the collection of giant viruses that they isolated from various environmental samples, such as soil, freshwater, seawater, and hypersaline soil and water [56]. This collection encompasses a wide range of mimiviruses. Thus, this team has accumulated the largest collection of giant viruses in the world and currently has published 59 strains of mimiviruses from the *Mimiviridae* family [54,55,56,57,58,59]. Three lineages, named A, B, and C, were described for amoebal mimiviruses [18,60], and distant mimiviruses having marine phagocytic eukaryotes as hosts were also described [25]. Like any other virus, virophages are obligate intracellular parasites, whose replication requires the metabolism and functions of the host cell but, in addition, concurrent infection by a giant virus. Sputnik virophage isolates have been isolated in association with mimiviruses belonging to lineage A and can grow easily with all mimiviruses from lineages B and C [26]. However, Zamilon is a unique virophage that grows with and parasitizes only lineage B and C mimiviruses, whereas mimiviruses of lineage A appear to be resistant [27]. Levasseur et al. recently described an adaptative viral defence system that represents a nucleic acid-based immunity against virophage infection [61]. Its discovery incepted from the search for a defence mechanism based on the genome integration of short DNA sequences from the invaders, by analogy with the prokaryotic CRISPR-Cas system of defence against viruses. Indeed, it was found that a 28-nucleotide-long sequence in the Zamilon genome was identical to sequences present in the genomes of lineage A mimiviruses, and 15-nucleotide-long sequences repeated four times were exclusively found in the genomes of lineage A mimiviruses (i.e., not in lineage B and C mimiviruses), and were linked to resistance and immunity to the Zamilon virophage [61]. These sequences were part of a genomic region harboring 27 genes, among which two were predicted to encode proteins with helicase and nuclease functions that could be involved in the degradation of foreign nucleic acids and have been validated experimentally. This defence system was, hence, described to rely on sequence-specific recognition of a short nucleic acid sequence, and was named the MIMIvirus VIrophage Resistant Element (MIMIVIRE). Although relying on the integration of DNA sequences from the viral invader, it is not homologous to prokaryotic CRISPR-Cas systems. Among differences between MIMIVIRE and CRISPR-Cas systems are the lack of proto-spacer adjacent motifs associated with the genomic repeats and of a strong homology with Cas-proteins [62]. It has also been hypothesized that this Zamilon virophage-resistant phenotype might rather rely on protein-protein interactions [62]. Further characterization of the MIMIVIRE system defence mechanism will now be allowed by additional biological demonstrations.

## 9. Abundance and Distribution of Virophages, and their Potential Impact on Virus-Host Ecology

During the past four years, metagenomic analyses have revealed the presence of sequences related to virophages in various environmental samples, from deep ocean to inland, and collected on different continents [63,30,47]. Virophages tend to be more frequently detected in freshwater and ocean sediments than deep seawater [30]. In addition, other virophage-related sequences have been detected in soil, glaciers, air on the east coast of Singapore as well as in the human gut, which suggests that virophages are common entities on Earth and even possibly present in humans [30,64]. In contrast with some obligate intracellular microorganisms that exist alone within their hosts, without any contact with other agents, virophages replicate sympatrically within amoebae with their associated giant viruses, and also with bacteria, fungi, or archaea, which can allow exchanges of sequences between these organisms [65,66]. The Sputnik virophage is able to impact on bacterial growth and regulate its host cell, through control of mimivirus virulence [67]. In view of the abundance of virophages in various and different environments, it has been suggested that they can play a role in regulating the dynamics of populations of giant viruses and their eukaryotic hosts, which may have a susbtantial influence on some ecosystems [28].

## 10. Virophages in Humans

The association of virophages with humans is still poorly known. Human exposure to giant viruses and to virophages should be concomitant. Senegalvirus, Shan virus, and megavirus LBA111 represent the first cases of isolation of giant viruses from human fecal or lung samples [57,58,65]. In addition, there has been an increasing body of data on the presence of giant viruses of amoeba in humans. Regarding virophages, Sputnik 2 was isolated from a contact lens rinse liquid, a human-associated sample [18], and a positive serology directed against the Sputnik virophage was described in two febrile patients, and seroconversion was observed in one of them [64]. In addition, during the past decade, metagenomic analyses have revealed the presence of numerous virophage-related sequences in human gut samples [30,45]. These data, which deserve to be bolstered, raise further questions about their potential pathogenicity.

## 11. Conclusions

Virophages are recently discovered viruses with an apparent broad worldwide distribution. Their discovery has occurred consequent to that of mimiviruses. They have contributed to differentiating mimiviruses from other viruses, bringing them closer to microbes. Indeed, they have revealed that mimiviruses, similarly to prokaryotes, could be infected by other viruses that integrate into their genome, and that mimiviruses have developed a mechanism of defense against these virophages. The virophage diversity will probably expand considerably in the near future and stimulate research to determine to what extent they infect giant viruses other than mimiviruses. The study of virophage origins, of their involvement in sequence exchanges between organisms, and of their impact on ecosystems is of substantial interest. Finally, based on preliminary findings, their association with humans warrants further investigation.

## Figures and Tables

**Table 1:** Main features of the virophages identified to date.

Virophage	GenBank Accession No.	Description Year	Source	Geographical Location	Associated Giant Virus (Taxonomy)	Protistan Host	Discovery Tool		Genome		Reference
								Size (bp)	Number of ORFs	G + C (%)	
Sputnik	EU606015	2008	Cooling tower water	Paris, France	Mamavirus	*Acanthamoeba polyphaga*	Culture	18,343	21	27	[17]
*Mavirus virophage*	NC_015230	2010	Coastal waters	Texas, USA	*Cafeteria roenbergensis virus*	Marine phagotrophic flagellate	Culture	19,063	20	30.3	[19]
OLV	HQ704801	2011	Organic Lake	Antarctica	*Organic Lake phycodnavirus* (distantly-related mimivirus)	Prasinophytes?	Metagenomics	26,421	24	36.5	[28]
Sputnik 2	NC_023846	2012	Lens liquid	Marseille, France	Lentillevirus	*Acanthamoeba polyphaga*	Culture	18,338	20	27	[18]
Sputnik 3	NC_023847	2013	Soil	Marseille, France	Mamavirus	*Acanthamoeba polyphaga*	Culture	18,338	20	27	[26]
*Phaeocystis globosa* virophage	NC_021333	2013	Dutch coastal waters	Southern North Sea	*Phaeocystis globosa virus* PgV-16T (Distantly-related mimivirus)	Algae	Genomics	19,527	16	35.8	[29]
YSLV1	KC556924	2013	Yellowstone Lake	USA	Phycodna- or mimiviruses	Microalgae?	Metagenomics	27,849	26	33.4	[30]
YSLV2	KC556925	2013	Yellowstone Lake	USA	Phycodna- or mimiviruses	Microalgae?	Metagenomics	23,184	21	33.6	[30]
YSLV3	KC556926	2013	Yellowstone Lake	USA	Phycodna- or mimiviruses	Microalgae?	Metagenomics	27,05	23	34.9	[30]
YSLV4	KC556922	2013	Yellowstone Lake	USA	Phycodna- or mimiviruses	Microalgae?	Metagenomics	28,306	34	37.2	[30]
ALM	KC556923	2013	Ace Lake	Antarctica	Possibly mimiviruses	Phagotrophic protozoa?	Metagenomics	17,767	22	26.7	[30]
Zamilon	NC_022990	2014	Soil	Tunisia	Mont1 virus (mimivirus)	*Acanthamoeba polyphaga*	Culture	17,276	20	29.,7	[27]
YSLV5	KM502589	2014	Yellowstone Lake	USA	Phycodna- or mimiviruses	Microalgae?	Metagenomics	29,767	32	51.1	[31]
YSLV6	KM502590	2014	Yellowstone Lake	USA	Phycodna- or mimiviruses	Microalgae?	Metagenomics	24,837	29	26.8	[31]
YSLV7	KM502591	2014	Yellowstone Lake	USA	Phycodna- or mimiviruses	Microalgae?	Metagenomics	23,193	26	27.3	[31]
Zamilon 2	N/A	2015	A non-aerated Bioreactor	USA	mimiviruses?	*Acanthamoeba* sp.	Metagenomics	6716	15 partial	32	[32]
DSLV1	KT894027	2016	Dishui Lake	China	N/A	Microalgae?	Metagenomics	28,788	28	43.2	[33]
QLV	KJ854379	2016	Qinghai Lake	China	N/A	N/A	Metagenomics	23,379	25	33.2	[34]

ALM, Ace Lake Mavirus; bp, base pairs; DSLV1, Dishui Lake virophage; N/A, not available; OLV, Organic Lake virophage; QLV, Qinghai Lake virophage; YSLV, Yellowstone Lake virophage.

## Box: Glossary

MIMIVIRE:	A mimivirus nucleic acid-based defense system against virophage infection.
Mobilome:	Mobile genetic elements in mimiviruses (including provirophages, transpovirons, and introns/inteins).
Polintons/Mavericks:	Large DNA transposons characterized by a unique set of proteins necessary for their transposition, including DNA polymerase B, a retroviral integrase, a cysteine protease, and an ATPase. Polintons are typically 15–25 kbp in length, and encode up to ten proteins.
Plasmid:	A DNA molecule that is physically separated from the genomic DNA and can replicate independently.
Provirophage:	A virophage genome integrated into the giant virus DNA.
Satellite virus:	A small subviral agent that depends on the presence of a helper virus for its propagation.
Transposon:	Linear DNA sequence of approximately 7 kbp that encodes six to eight proteins and is capable of moving independently within a genome. They are considered to be powerful motors of evolution and biodiversity.
Transpoviron:	Derived from “transposon”. It is a small linear transposable element detected in mimiviruses.
Virophage:	A viral parasite of giant viruses. It requires a giant virus factory co-infection for its replication.
